# Using Biopolymers to Control Hydraulic Degradation of Natural Expansive-Clay Liners Due to Fines Migration: Long-Term Performance

**DOI:** 10.3390/polym18020272

**Published:** 2026-01-20

**Authors:** Ahmed M. Al-Mahbashi, Abdullah Shaker, Abdullah Almajed

**Affiliations:** Bugshan Research Chair in Expansive Soils, Department of Civil Engineering, College of Engineering, King Saud University, Riyadh 11421, Saudi Arabia

**Keywords:** expansive-clay liners, biopolymers, hydraulic conductivity, flow, fines migration, long-term performance

## Abstract

Liners made of natural materials, such as expansive soil with sand, have a wide range of applications, including geotechnical and geoenvironmental applications. Besides being environmentally friendly, these materials are locally available and can be constructed at a low cost. The concern regarding these liners is sustainability and serviceability in the long run. The research conducted revealed significant degradation in hydraulic performance after periods of operation under continuous flow, which was attributed to the migration of fines. This study investigated the stabilization of these liners by using biopolymers as a cementitious agent to prevent the migration of fines and enhance sustainability in the long run. Two different biopolymers were examined in this study, including guar gum (GG) and sodium alginate (SA). The hydraulic conductivity tests were conducted in the laboratory under continuous flow for a long period (i.e., more than 360 days). The results revealed that incorporating biopolymers into these liners is of great significance for enhancing their sustainability and hydraulic performance stability. Further in-depth identification of the interaction mechanisms demonstrates that biopolymer–soil interactions create cross-links between soil particles through adhesive bonding, forming a cementitious gel that stabilizes fines and enhances the stability of the liners’ internal structure. Both examined biopolymers show significant stabilization of fines and stable hydraulic performance within the acceptable range, with high superiority of SA with EC20. The outcomes of this study are valuable for conducting an adequate and sustainable design for liner protection layers as hydraulic barriers or covers.

## 1. Introduction

In several geotechnical and geoenvironmental projects, liner layers are essential for controlling the flow of water to protect substructures (in highway, dams, and canals) or seepage of other hazardous leachates, toxic fluids, and industrial waste to subsoils and groundwater. Hydraulic barriers and protective liner layers made of expansive soils and sand have a wide application in such projects and facilities due to their natural compositions and eco-friendly nature (i.e., [1,2,3]). The effective role of these materials is their ability to swell, filling the voids between sand particles. This allows effective sealing, control of water hydraulic conductivity, and retention capacity of water or other harmful leachates or chemicals [4,5,6,7,8]. In addition, the economic feasibility of using locally available expansive soils with sand materials makes them an attractive alternative to commercial bentonite, particularly in arid and semi-arid regions where such materials are abundant (i.e., [7,9]).

Design of expansive clay liners requires great attention regarding the mechanical and hydromechanical behavior of these materials during drying and wetting conditions. The portion of these materials and their mineralogical composition is of great significance. Clay content significantly affects liner behavior by controlling hydraulic conductivity (permeability), strength, compressibility, and durability, with higher clay generally reducing permeability but increasing potential for shrink-swell and cracking, impacting long-term sealing performance, especially under freeze–thaw cycles or desiccation (i.e., [6,8,10]). On the other side, a higher portion of expansive clay also renders the liner more susceptible to volumetric changes and potential desiccation cracking if not adequately stabilized (i.e., [3,11]). Clay minerals provide self-healing and low-permeability properties, but excessive fines can lead to issues like particle breakage, while optimal clay content in sand mixtures enhances shear strength, but high content can reduce it (i.e., [12,13,14]). However, the efficacy of these liner layers is not solely determined by their initial state or performance but is critically defined by their long-term performance under varying environmental and operational conditions.

The most important point when designing liner layers is their sustainability and stability over their lifetime. However, the literature regarding the long-term performance of these liners under different environmental circumstances is scarce and requires further investigation (i.e., [11,15,16,17,18]). While initial laboratory tests may satisfy design requirements, the long-term performance of expansive clay–sand mixtures is a complex phenomenon in such materials and is characterized by extreme changes in hydraulic conductivity and structural integrity over time. A key study conducted by Almahbashi et al. [16] investigated the long-term efficiency of sand-clay liners under continuous water flow over periods exceeding 400 days and found significant fluctuations and eventual reduction in hydraulic conductivity due to initial fine migration and subsequent changes in the internal structure. Environmental and external circumstances (i.e., climatic changes and static and dynamic loads) that may these liners exposed to have a significant influence on their serviceability and lifetime. Al-Mahbashi et al. [17] showed that freeze–thaw cycles dramatically alter the hydraulic conductivity of expansive clay liners, with the magnitude of change heavily dependent on the mineralogical composition of the clay and the availability of moisture. Expansion of formed ice lenses during the freezing phase and vice versa during thawing significantly affect the internal structure and induce internal cracks. The intensity of these cracks is a function of the mineralogical composition of clay and is considered the main contributor to the deterioration of hydraulic performance (i.e., [8,19,20]). Al-Mahbashi and Alnuaim [18] postulated that dynamic loads simulating traffic or seismic activity, where properly designed and managed protective layers can reduce liner permeability by over six times under specific conditions. The majority of structural degradation and particle rearrangement occurs within the first 100 cycles of loading; the change in soil skeleton during these early stages of operation notably alters the hydraulic performance (i.e., [21,22]). Tran et al. [23] investigated the retention capacity of sandy soils after biopolymer treatment using Xanthan gum; the results revealed improvement in retention capacity and enhanced particle binding in sand.

The most common mechanism leading to the long-term degradation of the hydraulic performance of expansive clay liners is the migration of fine contents. Fines migration involves the detachment of clay particles or fines from the soil matrix under the influence of seepage forces, followed by their transport through the pore network of the liner body. Nasr-El-Din et al. [24] investigated this phenomenon in the context of reservoir engineering and found that in sand–clay mixtures, fines migration and clay swelling are the primary causes of long-term degradation. Al-Mahbashi et al. [16] provided robust insights into this behavior, finding that fine material migration leads to initial hydraulic conductivity fluctuations and the rearrangement of particles leads to final stabilization. Therefore, ensuring the sustainability of these liners requires a deep understanding of the degradation mechanisms that evolve over the service life of the infrastructure and the possible mitigation techniques. To this end, several investigations have been directed to enhance the stability of fins during the long time of operation, including chemical additives, mechanical improvement, and involving synthetic materials (i.e., [25,26]). Other studies have utilized advance of nanotechnology and polymers such as nano-silica particles to control the migration of fine particles from the body of liner layers (i.e., [15,27,28,29]). Taking into account the effect of manufacturing and using chemical additives or degradation of synthetic polymers into microplastics on the environmental changes, groundwater, and soil environment, the biopolymers are introduced as a sustainable, environmentally friendly alternative for soil stabilization.

While the immediate effects of biopolymers on soil strength, water retention capacity, permeability, and resilience have been well documented in the existing literature (i.e., [30,31,32,33,34,35,36,37,38]), their role in mitigating hydraulic degradation over the long-term remains unavailable or very rare. The interaction between biopolymers and expansive clays is particularly promising because the hydrophilic nature of many biopolymers allows them to bond with the clay surface water and enhance the stability of fine particles.

Recent numerical modeling studies integrate laboratory-derived parameters—such as the swelling index, macroscopic stability, stress–strain behavior, flow, shear strength, and ion-exchange capacity—into finite element and machine learning frameworks to predict long-term durability against leachate and environmental cycles. Other research demonstrates the effectiveness of biopolymers as sustainable landfill covers for mitigating fluid and gas transport (i.e., [39,40,41]). Collectively, the current study provides key long-term parameters and data that guide the efficient modeling and design of these barrier layers, which are still rarely up to date.

This study aims to investigate the stabilization of natural expansive clay liners using biopolymers as a cementitious agent to prevent the migration of fines and enhance their long-term stability, hydraulic performance, and sustainability. Two different biopolymers were examined in this study: guar gum (GG) and sodium alginate (SA). These biopolymers were selected for their proven adhesive properties and ability to form viscous gels in the presence of water. The hydraulic conductivity tests were conducted in the laboratory under continuous flow for an extended period, exceeding 360 days. Further in-depth assessment of soil-biopolymer interaction during this stabilization has been enhanced by representative models and microstructural analysis using scanning electron microscopy (SEM).

## 2. Materials and Methods

The study examined two different expansive clay liners, EC15 and EC20. The mixtures incorporated 15% and 20% natural expansive clay by dry weight for both liners, respectively, with the remaining portions being sand. Both sand and expansive clay have been locally sourced; the sand was obtained from the capital, Riyadh, and the expansive clay was sampled from the Eastern province, specifically Al Qatif city.

**Figure 1:**
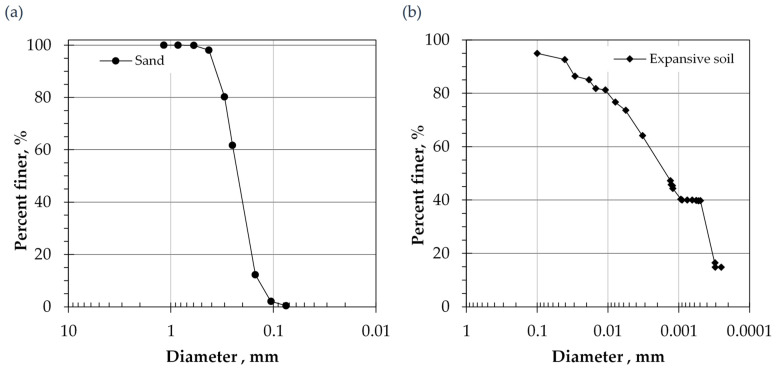
Grain size distribution for (**a**) sand, and (**b**) expansive clay.

The sand had a specific gravity of 2.67, determined according to ASTM D854 [42]. The particle size distribution was established in the laboratory via sieve analysis [43], as shown in Figure 1a. The resulting data indicated a uniformity coefficient of 1.745 and a coefficient of concavity of 0.945. This sand is classified as poorly graded sand (SP) according to the unified classification system ASTM D2487 [44].

Comprehensive geotechnical characterization of expansive clay soil was performed in the laboratory. The specific gravity of this clay was determined according to the specification described in ASTM D854 [42] and was found to be 2.7. The distribution of fines for expansive clay was conducted in the laboratory using a hydrometer test following ASTM D D7928 [45]. The gradation curve shown in Figure 1b; the portion of fine materials (clay) is about 56%. The investigations demonstrated a liquid limit of 160%, a plastic limit of 60%, and a shrinkage limit of 13%, determined in accordance with ASTM D4318 [46]. Based on these specific properties and following the Unified Soil Classification System, the soil is categorized as a high-plasticity clay [44]. Further specified identification regarding the mineralogical composition of this clay has been conducted utilizing XRD; the results revealed that the clay fraction contains a significant proportion of montmorillonite [47,48]. Consequently, the soil exhibits characteristic expansive properties, including a moderate-to-high swelling potential (18%) and a notable swelling pressure of 550 kPa [49]. A more comprehensive characterization of this soil’s hydromechanical behavior can be found in Al-Mahbashi et al. [16].

The biopolymers utilized in this study were sodium alginate (SA) and guar gum (GG), with a dosage of 3% of the dry weight. The selection of these biopolymer types was based on their sustainability, availability, thickening and film formation, gelling capability, and simplicity of use. Furthermore, this percentage of biopolymers was proven to have a significant effect on permeability, retention capacity, stability, and shear strength for the treated soils (i.e., [36,50,51]). Sodium alginate is a linear polysaccharide derived from alginic acid that consists of α-l-guluronic (G) and 1,4-β-d-mannuronic (M) acids. Notably, sodium alginate—a component found in the cell walls of marine brown algae—contains between 30% and 60% alginic acid. Guar gum is a natural thickening agent derived from guar beans. More details about these biopolymers and their composition are available in the literature (i.e., [52,53,54]). Regarding the cost of biopolymers as an environmentally friendly alternative compared to traditional stabilizers, the difference is not significant. As a specific case study, Turrakheil et al. [55] reported the comparison between guar gum biopolymer and cement for soil treatment. Guar gum costs approximately GBP 24/m^3^ compared to roughly GBP 15/m^3^ for cement. On the other hand, the total CO_2_ emissions for treating 1.0 m^3^ of soil are 86.4 kg for cement compared to 1.6 kg for guar gum biopolymer (i.e., IEA [56]).

## 3. Mixtures and Specimens Preparation

The wet mixing technique was used in this study; the distilled water was mixed with the biopolymer in a mechanical mixer for 20 to 60 min to ensure a homogeneous solution. The speed of the mixer was within the range of 1500 RPM, and the dissolution took place at a room temperature of 25 ± 1.5 °C. The biopolymer was added in small quantities during the mixing process to prevent any formation of dry biopolymer lumps. At these dissolution conditions, the required viscosity of tested biopolymers could be achieved for better stabilization performance, including bio-clogging or pore filling, cross-linking, and mechanical bonding (i.e., [57,58]). After that, this solution was added to the soil mixture in small amounts and mixed thoroughly. The mixtures were conditioned (mellowed) with a predetermined amount of water corresponding to their respective optimum moisture contents, established via standard compaction curves as defined by ASTM D698 [59].

Identical specimens were then compacted to their corresponding maximum dry densities within an 80 mm diameter and 40 mm height mold using a hand-operated jack.

The selection of the sample size was primarily guided by the requirement for a controlled environment to establish baseline hydraulic performance under specific variables. ASTM D5856 [60] specifically notes that flow patterns in small laboratory specimens may not always match large field scales, but they provide a standardized, cost-effective diagnostic tool for evaluating materials before large-scale development. The sidewall effect, expected fissures, and dissection cracks were reported as key factors affecting the large-scale field results. In this study, the presence of swelling materials (expansive soil) minimizes the effect of side-wall leakage, and under rewetting conditions in the field, the effects of desiccation cracks may be mitigated by the soil’s swelling potential. In addition, laboratory samples are often more homogeneous than field-compacted layers, potentially leading to hydraulic conductivity measurements that are more conservative than those observed in situ by one to three orders or more (i.e., [61,62]).

Jafari et al. [63] examined the impact of the size effect for specimens during hydraulic conductivity measurements; the results revealed that for diameters larger than 2 inches (i.e., 5.0 cm), the difference was insignificant. In the field, the size effect is minimal in low-permeable materials such as expansive clay amended liners (i.e., [64]).

## 4. Testing Procedures

**Figure 2:**
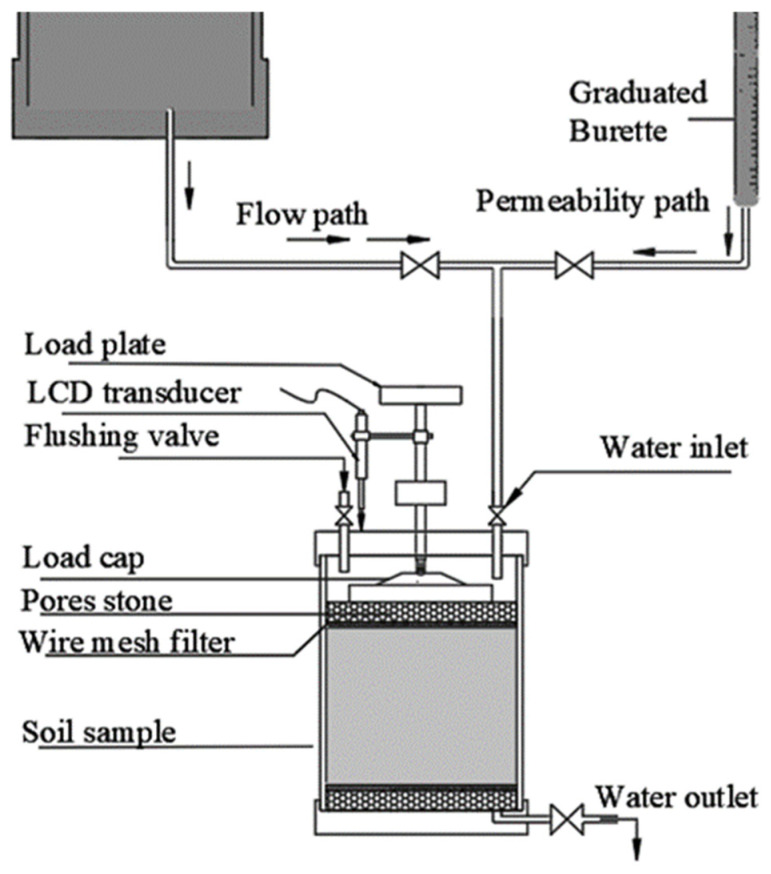
Schematic diagram of the testing system.

The main test performed in this study is hydraulic conductivity (k). The test commenced with a combination of continuous flow. Prepared mixtures of sand-expansive clay before and after treatment with biopolymers SA and GG were compacted to the maximum dry density as per the compaction curve. As shown in the schematic diagram of the testing system (Figure 2), the permeameter cell mainly consists of a rigid-wall mold, top and bottom plates, and regulator valves. The specimens were compacted inside the cell mold, 80 mm in diameter and 40 mm in height, and an appropriate recess was maintained to accommodate the porous stones on the top and bottom sides of the device cell, as shown in Figure 2. The upper side of the specimen inside the cell was connected via a rod that penetrated the upper plate. An LCD transducer was attached to this road and connected to a computer via software. The specimen was initially saturated using distilled water under a small pressure of 5 kPa; the deformation during this stage was monitored through the attached transducer. This process takes about 5 to 7 days, and saturation was achieved when water started to flow out of the specimen. As shown in the schematic diagram of the testing system, two separate paths were designed, the first one to provide a continuous flow through the attached water tank. The other path connected to the graduated burette to conduct the falling head hydraulic conductivity test, following the designation described in ASTM D5856 [60]. The formula used for calculating hydraulic conductivity in this method is presented in Equation (1). According to this method, when four successive measurements for hydraulic conductivity with minimal variation are attained (±25% of the mean value for *k* ≥ 1 × 10^−10^ m/s), the system reaches a stable condition (steady flow), and long-term measurement can be performed. The measurement of hydraulic conductivity was performed 3 to 9 times per day, head variation (*h*_1_ and *h*_2_) was recorded, and the coefficient of permeability was calculated as shown in Equation (1).

(1) $$k=\frac{a\;L}{2\;A\;t}\ln\left(\frac{h_{1}}{h_{2}}\right)$$

where

*k* = hydraulic conductivity, cm/s;

*a* = cross-sectional area for the graduated burette, cm^2^;

*L* = length of specimen, cm;

*A* = cross-sectional area for specimen, cm^2^;

*t* = elapsed time between the determination of heads h_1_ and h_2_, s;

*h*_1_ = the head loss across the specimen at time *t*_1_, cm;

*h*_2_ = head loss across specimen at time *t*_2_, cm.

## 5. Results and Discussion

The results obtained from the experimental program conducted in this study are presented in these sections.

### 5.1. Degradation of the Liner’s Efficiency over Time

**Figure 3:**
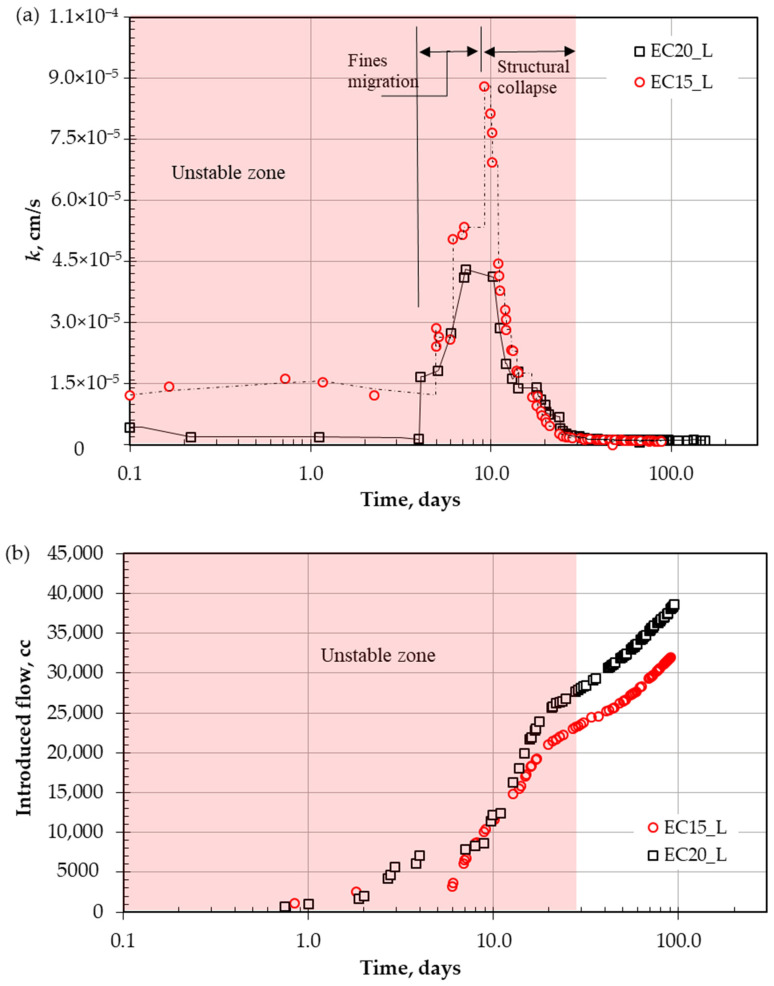
Unstable zone for tested liners: (**a**) hydraulic conductivity, and (**b**) introduced flow.

Figure 3 shows the hydraulic conductivity variation for both liners, EC15 and EC20, over the extended period of testing. In the initial stage of tests conducted in accordance with ASTM D5856 [60], measurements start when the first three successive measurements show marginal variance, and continuous flow is guaranteed during the entire experimental period. The shaded area in the chart represents the unstable zone. After the first three days, the hydraulic conductivity starts to deteriorate and reaches its highest peak, increasing by more than six times on the ninth day. The plausible interpretation of this deterioration is the migration of fine contents with the introduced flow (this zone is defined in Figure 3a).

**Figure 4:**
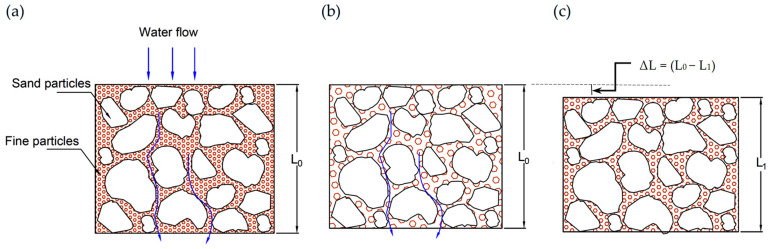
Representative cross-section for (**a**) EC liner’s initial state, (**b**) fine migration during flow, and (**c**) particle rearrangement and skeleton adjustment for final stabilization.

A combined representative pattern showing the cross-section of these liners during different stages has been established in Figure 4a–c. Figure 4a shows a representative cross-section demonstrating sand particles, fine particles filling the voids, and water flows through the section. With the introduction of flow, the fines start migrating from the voids, causing more open channels. This is attributed to the fast degradation in permeability at the first stage (i.e., fines migration). Fine migration from clay liners due to water flow is a complex process where fine clay particles detach and move within the soil matrix, potentially causing significant issues such as permeability degradation and internal erosion. The sharp degradation in *k* could lead to a failure condition in the field; however, this risk should be evaluated against the expected flow and long-term performance data established under laboratory conditions.

**Figure 5:**
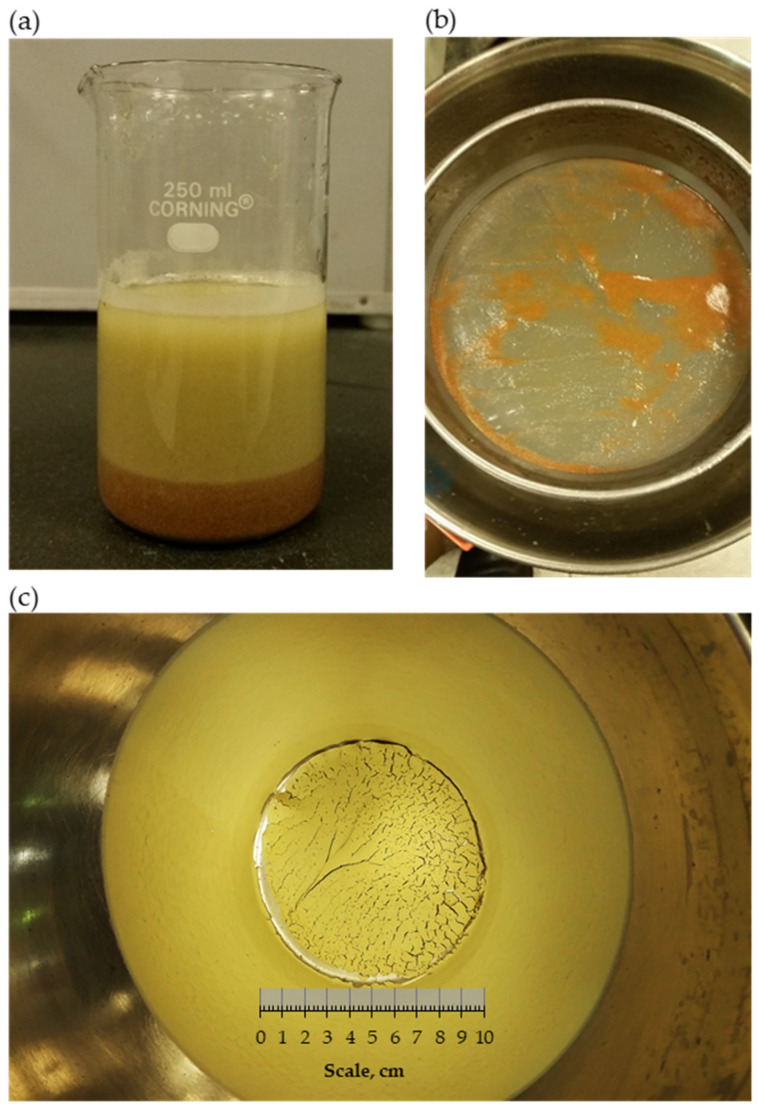
Determination of fine content by the washing technique: (**a**) specimen soaked in distilled water, (**b**) materials washed over a #200 sieve, and (**c**) fines passed after oven-dry.

**Figure 6:**
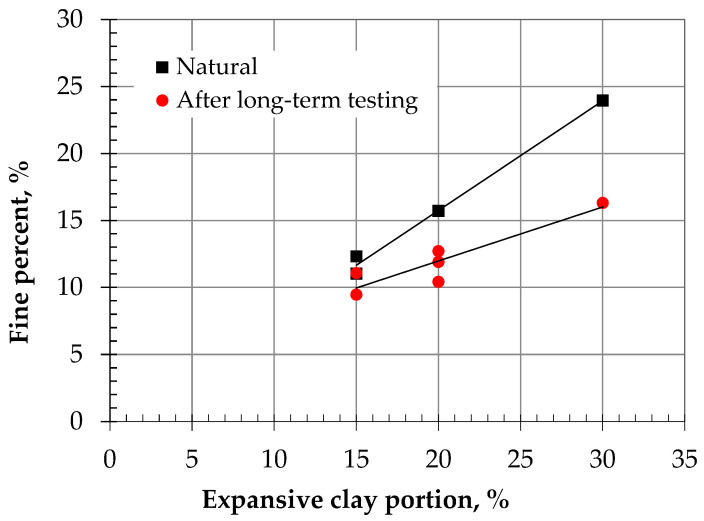
Measured fines before and after conducting long-term performance in different expansive clay liners.

For convincing quantification of the migration of fines through the body of expansive clay liners, the content of fines (material finer than 75-μm) was measured for different liners containing 15%, 20%, and 30% portions of expansive clay following the standard described in ASTM D1140 [65]. The tests were conducted on specimens of these liners before the test (i.e., natural) and after conducting the long-term hydraulic conductivity test with continuous flow. The specimen was submerged in water for several days and blended gently to separate the clay fines from sand particles, as shown in Figure 5a. Then, using distilled water, the materials were washed over a #200 sieve (Figure 5b). Afterward, the fines passed through water were oven-dried (Figure 5c), and the percentage of fines was calculated. Figure 6 shows the trend of results obtained from these tests. The results indicate that a considerable number of fines migrate during the test. In the presence of clay and biopolymers, the bonding and crosslinking prevent detachment and skeleton alteration to free sand as occurred under high pressure in pure sands (i.e., [66,67]). The percentage loss of fines shows 16%, 20%, and 32% for expansive clay portions of 15%, 20%, and 30%, respectively. Fine migration is also shown in the expansive clay portion and increases with the increase in this percentage. Al-Mahbashi et al. [16] also reported similar behavior for different liners before and after a long period of testing.

As small clay particles continue to escape, the soil’s structural composition and internal structure weaken with more pore network channels, as shown in Figure 4b. The degradation reached a peak with an increase in permeability of more than 6 orders of magnitude on the sixth day of continuous flow.

At this end, the weakened internal structure collapses, as shown in Figure 4c (ΔL = L_0_ − L_1_), and the fine particle accumulation in pore spaces causes clogging again. This mechanism takes a period and could be repeated for several peaks until reaching a stable structure and performance. During the re-clogging of pores, the permeability gradually reduced back as shown in Figure 3a, and within 40 days, approximately reached the initial value before the degradation process. Afterwards, the performance stabilized on the 40th day, where the change was marginal within the variation of ±25% of the mean value [60], and the trend shows a difference in less than ±5% between successive records.

### 5.2. Effect of Biopolymer on the Long-Term Performance of Tested Liners

**Figure 7:**
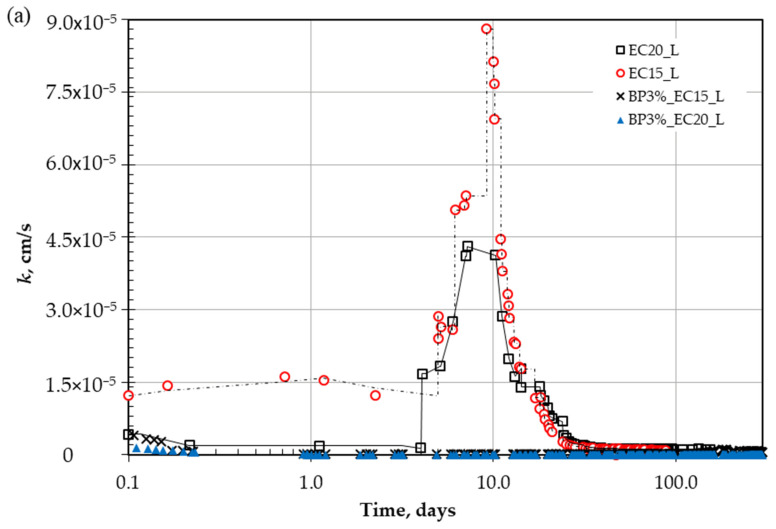

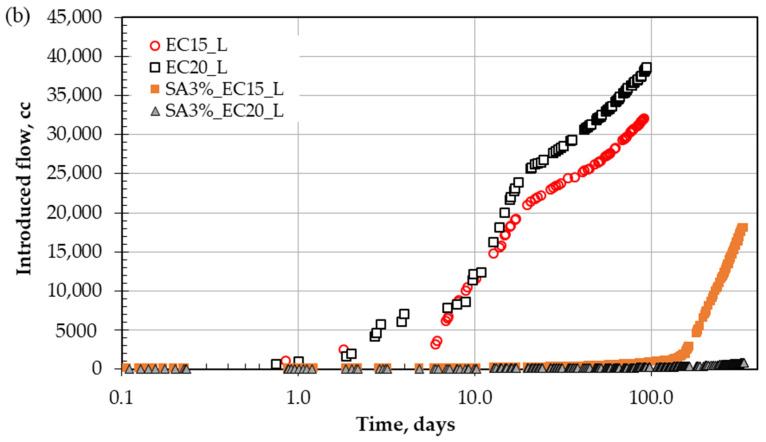
Effect of biopolymer treatment on (**a**) hydraulic conductivity; (**b**) cumulative flow of tested liners.

Referring to the previous discussion regarding the deterioration in hydraulic conductivity in tested liners, this section presents the effect of incorporating 3% biopolymer into both liners, EC150 and EC20, over an extended period of one year, as shown in Figure 7a,b. Figure 7a shows the permeability versus the time of introduced flow, and Figure 7b shows the cumulative flow over the testing period. For the sake of comparison, the variation in hydraulic conductivity for liners before biopolymer stabilization was depicted in the same figures as dotted lines. The results show a significant alteration in the performance of liners during the extended period of testing. The hydraulic conductivity shows a stable trend over time, and no notable degradation (i.e., sharp peak) was noticed compared to the natural liners (Figure 7a) in the first days (i.e., 10 days). In general, biopolymers stabilize soil through a combination of physical, chemical, and microstructural mechanisms that bind soil particles together, reduce permeability, and improve overall stability of liners (i.e., [68,69,70]). The type of biopolymer determines the stabilization and reaction mechanisms, besides the type of stabilized soils. In this study, the used biopolymers are both polysaccharide biopolymers, classified as complex carbohydrates, and produce advanced composite films or hydrogels after hydration.

### 5.3. Comparing the Effect of Biopolymer Type on Long-Term Performance

**Figure 8:**
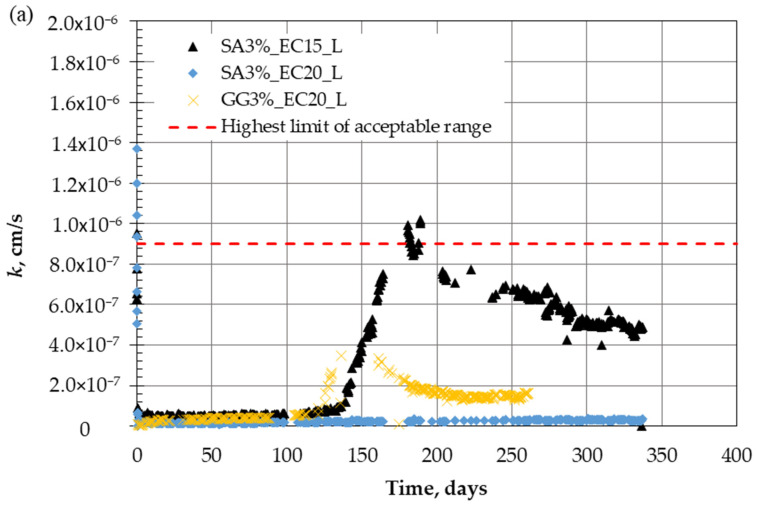

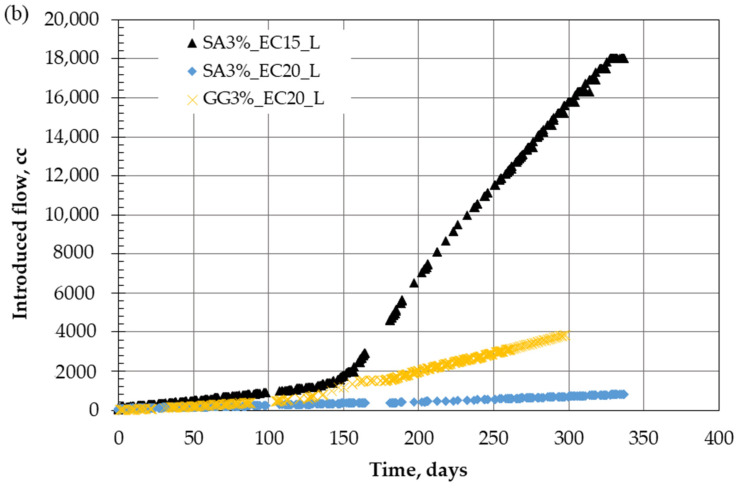
Comparison of different biopolymers on the long-term performance of (**a**) hydraulic conductivity, and (**b**) introduced flow.

To assess the role of biopolymer types in different expansive clay liners, Figure 8a compares the long-term hydraulic conductivity at a large scale for SA and GG used in the stabilization of both ECL15 and ECL20 liners. In addition, Figure 8b shows the accumulated flow during the testing period. The hydraulic conductivity exhibits a stable trend for both biopolymers in both liners over the first 120 days.

A small peak was noted after that for EC15 treated with SA, the hydraulic conductivity rose to the highest limit of the acceptable range (9 × 10^−7^ cm/s). It is worth noting that these values are still in the acceptable range for a wide application of liners. The liner of EC20 treated with 3% GG also shows a jump in hydraulic conductivity up to 4 × 10^−7^ cm/s at 150 days of continuous operation, and this is inside the acceptable range. However, the specimen of EC20 liner treated with 3% SA shows a stable trend over the entire period of testing. The variations in (*k*) and introduced flow for SA-treated specimens were modeled using a polynomial function that provided the best fit across different operating periods. These models yielded the highest coefficients of determination (*R*^2^), as detailed in Equations (2)–(5) for EC15_Land CE20_L, respectively. Standing on the mechanisms and interaction between soil-biopolymers, improvement could be achieved by the following key mechanisms (i.e., [35,68,70,71,72,73,74,75]): (1) inter-particle cohesion and pore structure alteration, biopolymers filling the pores and acting as a binder between particles. (2) Hydrogel components produced after hydration initiate a gel matrix surrounding particles; in addition, strong hydrogen bonds are developed between clay particles and biopolymer chains. (3) The cross-linking mechanism also provides strong networks that resist erosion of fine particles.

Sodium alginate and guar gum biopolymers primarily improve soil properties by forming viscous hydrogels that promote the agglomeration and bonding between fine particles, and lead to a more stable internal structure. Hence, the mechanical behavior of biopolymer-treated soil depends on the formation of soil–biopolymer matrices. The high efficiency to provide stable hydraulic performance over time achieved by SA is attributed to the anionic linear polysaccharide nature of this biopolymer, which provides effective gelling and cementitious mechanisms.

The recent mechanisms could be explained by highlighting the differences in binding efficiency between SA and GG with clay particles; these differences are primarily driven by their distinct ionic charge characteristics, which dictate whether they interact via electrostatic forces or hydrogen bonding. SA is negatively charged with carboxyl groups, and at neutral pH, these groups are partially ionized, giving it a high net negative charge and inducing cationic bridging with the cations between clay particles. While GG has a non-ionic nature of galactomannan polysaccharide, lacks charged functional groups and primarily interacts through its abundant hydroxyl groups (hydrogen bonding). Further in-depth discussion regarding the possible interaction mechanism between the clay particles and biopolymer, with a schematic diagram including cation bridging and hydrogen bonding are available in the documented literature [35,41,76,77,78,79,80,81,82].

**Figure 9:**
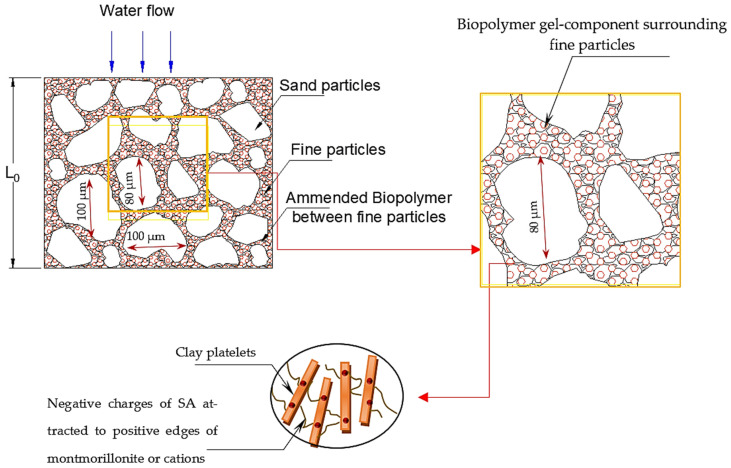
Representative cross-section showing interaction of biopolymer–clay particles in the voids between sand particles.

Sodium alginate forms a gel-like substance when mixed with water. Its key feature is the ability to form strong, stable calcium alginate (Ca-alginate) gels through ionic cross-linking with multivalent cations like calcium or sodium ions naturally present in the expansive soil (i.e., montmorillonite). The cross-section shown in Figure 9 shows the network bonding between fine particles due to these interaction mechanisms.

(2) $$k_{cm/s}=\left\{\begin{array}{c} 6\times 10^{-12}t^{2}-4\times 10^{-10}t+6\times 10^{-8},\;\;1<t\leq 136\;\mathrm{days},\;\;R^{2}=0.8022\;\\ -1\times 10^{-10}t^{2}+6\times 10^{-8}t-6\times 10^{-6},\;\;130<t\leq 188\;\mathrm{days},\;\;R^{2}=0.9506\\ 9\times 10^{-15}t^{4}-1\times 10^{-11}t^{3}+\\ 4\times 10^{-9}t^{2}-7\times 10^{-7}t+4\times 10^{-5},\;\;188<t\leq 336\;\mathrm{days},\;\;R^{2}=0.8022\;\;\;\;\;\;\;\; \end{array}EC15\_L\right.,$$

(3) $${flow}_{{cm}^{3}}=\left\{\begin{array}{c} 0.0103t^{2}+7.036t+109.480,\;\;1<t\leq 136\;\mathrm{days},\;R^{2}=0.9959\;\;\\ 0.002t^{2}+89.345t-11391,\;\;\;136<t\leq 336\;\mathrm{days},R^{2}=0.9974\;\;\; \end{array}\right.\backslash EC15\_L$$

(4) $$k_{cm/s}=3\times 10^{-18}t^{3}-4\times 10^{-14}t^{2}+7\times 10^{-11}t+1\times 10^{-8},\;1<t\leq 336\;days,\;\;\;\;R^{2}=0.8861\;\;\;\;\;\;\;\;EC20\_L$$

(5) $${flow}_{{cm}^{3}}=5\times 10^{-5}t^{2}+2.129t+50.828,\;1<t\leq 336\;days,\;\;\;\;R^{2}=0.9959\;\;\;\;\;\;\;\;EC20\_L$$

**Figure 10:**
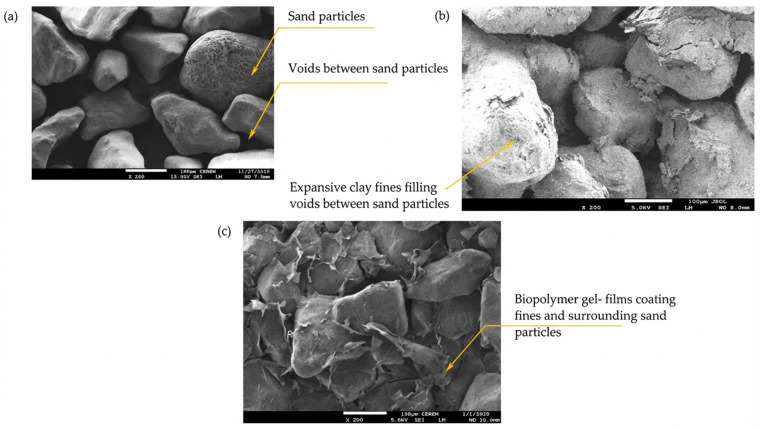
Microstructural study conducted by SEM for (**a**) sand particles, (**b**) clay filling the voids between sand particles, and (**c**) the presence of amended biopolymer SA.

The microstructural investigation utilized Scanning Electron Microscopy (SEM), as shown in Figure 10a–c. These figures illustrate three distinct conditions: (a) initial conditions showing native sand particles and interstitial voids; (b) voids between sand particles filled with expansive clay; and (c) the final case, featuring the presence of a sodium alginate (SA) biopolymer. The tests have been carried out after compaction and a mellowing period for treated specimens (i.e., 48 h); the specimens were freeze-dried before commencing the test to eliminate the disturbance of internal structure due to oven drying shrinkage. The SEM images for the treated samples reveal that the biopolymer forms gel films that coat the soil particles. This coating induces tightened bonds and creates a more stable, cohesive soil matrix. A key benefit of the inherent viscosity of these biopolymer products is their superior ability to reduce the migration of fine particles [70,83]. Recent studies [36,51] also reported the efficiency of SA (2–3%) to reinforce natural soils via the formation of soil-biopolymer matrices. It could be inferred from the preceding discussion that a 3% concentration of SA is sufficient to maintain the stable hydraulic performance of the EC20 liners. Furthermore, incorporating a low percentage of expansive clay is highly significant, as it effectively minimizes the formation of drying cracks and mitigates massive volume changes typically associated with wetting conditions.

## 6. Summary and Conclusions

The study has been conducted to investigate the efficiency of selected biopolymers in controlling the deterioration of hydraulic performance due to fines migrations for expansive clay liners in the long-term of serviceability. Continuous flow was applied during the test period, which was extended for more than a year. The following are the main conclusions:

- The hydraulic conductivity of the examined expansive clay liners undergoes extreme degradation under continuous flow, and an unstable zone was extended over the first forty days.
- The degradation of hydraulic conductivity is attributed to the migration of fine particles; the measured percent loss of fines varied from 16% to 32% and showed an ascending trend with the increase in expansive clay portions.
- Fine migration leads to a sharp peak permeability increase exceeding six times the initial value within the first ten days; subsequently, the weakened structure collapses, which enables particles rearrangement and a recovery of hydraulic conductivity to a stable performance level after forty days.
- Incorporating a 3% biopolymer (SA and GG) significantly enhanced the long-term stability of the hydraulic conductivity for both EC150 and EC20 liners. Unlike untreated ones, the biopolymer-treated liners maintained a stable trend over an extended one-year testing period.
- Specifically, polysaccharide biopolymers used in this study stabilize soil by binding particles together through physical, chemical, and microstructural mechanisms. The formation of advanced composite films or hydrogels significantly enhances particles bonding and improves overall stability.
- EC20 liner treated with 3% SA showed the most stable performance. Microstructural analysis confirms biopolymer gel films coat particles, creating a stable matrix and minimizing fine particle migration, and a 3% SA concentration is sufficient for stable hydraulic performance in EC20 liners, while low expansive clay content minimizes drying cracks and volume changes.

**Limitations of the study:** The research focused on the long-term performance of hydraulic conductivity for natural expansive clay liners and degradation induced due to fines migration. The tests were carried out for laboratory specimens under continuous flow. Two biopolymers, sodium alginate (SA) and guar gum (GG), were used as cementitious agents to eliminate fine migration and consequently provide a stable and sustainable performance. Further future work is recommended under different field circumstances (i.e., wetting/drying, and freeze–thaw).

## Data Availability

The original contributions presented in this study are included in the article. Further inquiries can be directed to the corresponding author.
